# Graduates in Rush Medical College—Session of 1849–50

**Published:** 1850-05

**Authors:** 


### ARTICLE III.

### GRADUATES IN RUSH MEDICAL COLLEGE—SESSION OF 1849–50.

At the Annual Commencement of Rush Medical College, held Thursday, Feb. 7th, 1850, the degree of Drctor of Medicine was conferred on the gentlemen, whose names, with the titles of their Inaugural Theses, are annexed:

| *Name.* | *Residence.* | *Title of Theses.* |
|---|---|---|
| J. L. Anderson, | Illinois, | Fever. |
| Cyrus G. Blood, | Iowa, | Circulation of the Blood. |
| Clay Brown, | Indiana, | Ophthalmia. |
| Henry F. Brown, | Illinois, | Depression and Depressing Agents, |
| William F. Coleman, | " | Dyspepsia. |
| Kimball Favor, | " | Materia Medica. |
| Edward J. French, | " | Pneumonia, |
| John Gregory, | " | Hydropathy. |
| Samuel Rush Haven, | " | Free Medical Schools. |
| Isaiah P. Hamilton, | Wisconsin, | Cause & Effect of Marsh Miasmata. |
| George Higgins, | " | Cholera. |
| Orson C. Hoyt, | New-York, | Cholera. |
| Alexander Hull, | Illinois, | Miasmata. |
| Franklin B. Ives, | " | Typhoid Fever. |
| Thomas G. Klepper, | Minnesota, | Fever. |
| M. Tevis Klepper, | Illinois, | Remitting Fever. |
| Charles J. Macon, | Iowa, | Tetanus. |
| Manly Miles, Jr., | Michigan, | Pathological Changes of the Blood. |
| Risdon C. Moore, | Indiana, | Cholera Infantum. |
| Alonzo L. McArthur, | Illinois, | Etiology and and Pathology of Phthisis Pulmonalis. |
| William C. Oatman, | " | Chymification. |
| Silas S. Parkhurst, | Michigan, | Scrofula. |
| William J. Paugh, | Illinois, | Pneumonia Biliosa. |
| William W. Perry, | Michigan, | Peritonitis. |
| John M. Phipps, | Indiana, | Cholera. |
| Giles P. Ransom, | Illinois, | Pathology of Fever. |
| David Rogers, | " | Pernicious Fever. |
| Josiah R. Snelling, | Ohio, | Science of Medicine. |
| John W. Spaulding, | Illinois, | Typhoid Fever. |
| Benjamin G. Stephens, | " | The Influence of Correct Theories in Medicine. |
| Benjamin F. Stephenson | Iowa, | Hepatitis. |
| Edwin Stewart, | Michigan, | Inflammation, |
| Isaac E. Thayer, | Wisconsin, | Character and Duty of a Physician. |
| John M. Todd, | " | Cholera. |
| Henry D. C. Tuttle, | Michigan, | Neuralgia. |

| *Name.* | *Residence.* | *Title of Theses.* |
|---|---|---|
| James P. Walker, | Illinois, | Bilious Remitting Fever. |
| Harmon Wasson, | " | Epidemic Cholera. |
| George S. Wheeler, | " | Hematemesis. |
| Zachariah H. Whitmire, | " | Fever. |
| Thomas Wilkins, | " | Treatment of Diseases in the West. |
| Wm. W. R. Woodbury, | " | Pneumonia. |
| James R. Zearing, | " | Ophthalmia. |

The Honorary Degree of Doctor of Medicine was conferred on Dr. E. S. Cooper, of Henderson, Ill., and the *ad eundem* Degree on James S. Whitmire, M. D., of Metamora, Ill.

